# Transdermal Delivery of Indirubin-Loaded Microemulsion Gel: Preparation, Characterization and Anti-Psoriatic Activity

**DOI:** 10.3390/ijms23073798

**Published:** 2022-03-30

**Authors:** Enxue He, Hailing Li, Xiaokun Li, Xunxun Wu, Kun Lei, Yong Diao

**Affiliations:** 1 School of Biomedical Science, Huaqiao University, Quanzhou 362021, China; heexue@stu.hqu.edu.cn (E.H.); lihailing925@163.com (H.L.); 18014071010@stu.hqu.edu.cn (X.L.); wuxunxun2015@163.com (X.W.); 2 School of Medical Technology and Engineering, Henan University of Science and Technology, Luoyang 471023, China

**Keywords:** indirubin, microemulsion, hydrogel, psoriasis, transdermal delivery system

## Abstract

Psoriasis is an immune disease caused by rapid and incomplete differentiation of skin basal cells. Natural products such as indirubin have historically served as excellent sources for the treatments of psoriasis. However, the poor solubility and bioavailability due to its plane and rigid crystal structure, which limits its efficacy. Herein, to improve the efficacy of indirubin, a hydrogel-based microemulsion drug delivery system was developed for transdermal delivery. The mean droplet size of the optimized microemulsion was 84.37 nm, with a polydispersity index (PDI) less than 0.2 and zeta potential value of 0~−20 mV. The transdermal flux and skin retention of indirubin at 24 h were 47.34 ± 3.59 μg/cm^2^ and 8.77 ± 1.26 μg/cm^2^, respectively. The optimized microemulsion was dispersed in carbomer 934 hydrogel to increase the consistency. The indirubin-loaded microemulsion gel was tested on an imiquimod-induced psoriasis mouse model. Results showed that this preparation can improve psoriasis symptoms by down-regulating the expression of IL-17A, Ki67, and CD4^+^T. This experiment provides great scalability for researchers to treat psoriasis, avoid first-pass effects, and increase the concentration of targeted drugs.

## 1. Introduction

Psoriasis is a chronic inflammatory skin disease occurring worldwide [1]. Skin cells renew about every four weeks in healthy people, while skin cells rapidly grow and migrate to the epidermis within several days forming thick skin patches called squamous silvery patches in psoriasis patients [2,3]. Depending on the severity, the treatment of psoriasis includes local treatment, phototherapy, and systemic drug therapy. Local treatment with creams and ointments containing corticosteroids or vitamin A/D analogues is effective for mild to moderate psoriasis [4,5,6]. With continued use of the drug, the skin becomes resistant to various treatments, and psoriasis often recurs periodically [7]. However, due to the low efficacy and despite fewer side effects, these treatments are unsatisfactory. Therefore, the effective treatment of psoriasis is still a challenging topic, and it is necessary to develop alternative treatment methods.

Natural products have historically served as excellent sources of active compounds for alleviating psoriasis. With the advantage of being generally safe and having few side effects, great attention has been paid to natural products in the past few decades [8,9]. Indirubin (IR), isolated from indigo naturalis, has been shown to improve psoriasis without causing any serious adverse events [10,11]. Previous studies have shown that IR could alleviate psoriasis via regulation of keratinocyte proliferation and reduces inflammatory processes [12]. Clinical studies have proved that IR ointment is an effective method in the treatment of psoriasis [11,13]. Mechanistic studies revealed that local application of IR can improve psoriasis symptoms without causing any serious adverse events. For example, IR can inhibit keratinocyte proliferation by inhibiting the activation of EGFR. By up-regulating the mRNA and protein expression of Claudin-1, the function of tight junctions in human primary keratinocytes was improved. Inhibition of the TAK1 signaling pathway attenuates the expression and production of IL-17A-induced CCL20 in keratin-forming cells. Psoriasis symptoms were improved by down-regulating proliferation marker PCNA expression and up-regulating protective skin protein expression. IR also improved the inflammatory severity of imiquimote-induced psoriatic skin in mice by inhibiting γδT cell-mediated inflammatory response, and in this model, local application of IR proved to be effective and safe [12,13,14].

However, the low solubility and bioavailability limits the effect and application of IR [14]. Nanocarrier-based local drug delivery systems, such as increasing the solubility of hydrophobic drugs and improving drug permeability, were established to overcome these shortcomings [15,16]. Nevertheless, people with psoriasis have lower levels of ceramides, higher cholesterol levels, and an over-proliferation of keratinocytes, leading to an increase in scaly plaques that make the skin thicker and harder [17]. Therefore, how to effectively deliver IR analogs to the deep skin of psoriasis is a great challenge. Currently, several drug carriers have attempted to increase percutaneous penetration, such as microemulsion (ME), nanoparticles, and nanosuspensions [18]. ME has been widely used for transdermal drug delivery due to their remarkable penetration-enhancing effect [19,20]. ME refers to a thermodynamically stable system consisting of an oil phase, aqueous phase, surfactants, and co-surfactants. ME can be divided into three types of o/w, w/o and bicontinuous phase, among which the o/w type ME has a strong solubilizing effect on drugs with poor water solubility [21,22]. The ME has the advantage of high moisturizing and permeability, and forms a drug depot in the skin after transdermal administration so as to realize the effective penetration of the drug. In addition, ME diameters are usually small in size, which makes them a suitable vehicle for drug delivery [18]. Therefore, ME has great potential for skin drug delivery.

**Figure 1:**
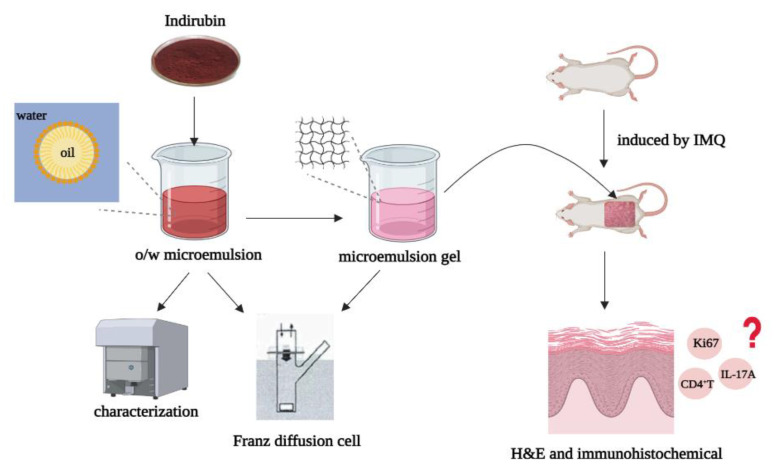
The schematic illustration of the study on indirubin microemulsion gel. Firstly, indirubin microemulsion was prepared and characterized, then carbomer 934 was used as a gel matrix to increase the viscosity of the microemulsion, and the anti-psoriatic activity of the preparation was evaluated by H&E and immunohistochemical assays.

The present study intended to prepare and evaluate a microemulsion-based hydrogel as a transdermal delivery carrier for IR. Herein, IR-loaded ME gel was prepared and characterized. In addition, its anti-psoriasis effect was demonstrated by in vitro transdermal tests and in vivo animal experiments. Therefore, this study is expected to lay a good theoretical and application foundation for the preparation of stable and transdermal IR-ME gel formulations. The experimental flow chart is shown in Figure 1.

## 2. Results

### 2.1. Preparation of IR-ME and IR-ME Gel

#### 2.1.1. Solubility Study and Selection of Adjuvants for ME System

**Figure 2:**
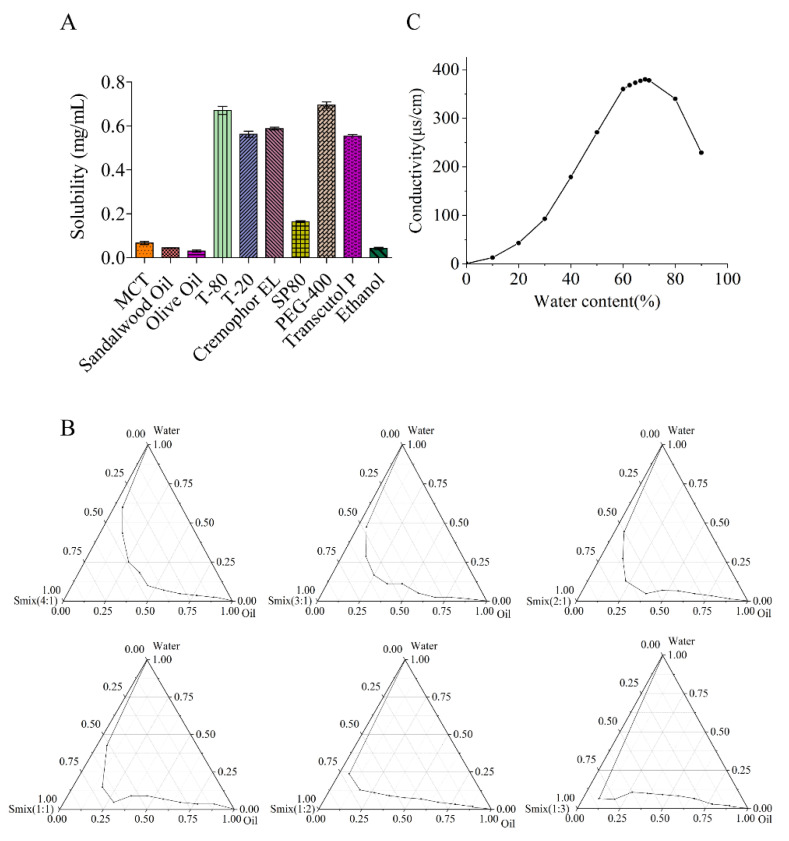
Preparation of IR-ME. (**A**) IR solubility in different oils, surfactants, and co-surfactants (*n* = 3); (**B**) pseudo-ternary phase diagrams of the MCT/Cremophor EL/PEG-400/water system; (**C**) the curve of ME conductivity with water content.

Different oil phases, surfactants, and co-surfactants have great influence on the formation of ME. Surfactant and co-surfactant are significant for droplet stability by reducing surface tension [23]. Herein, suitable adjuvants for the composition of the ME system were screened. The solubility of IR in various oil phases, surfactants, and co-surfactants were investigated (Figure 2A). Of the oil phases listed, IR had the highest solubility in MCT (0.067 ± 0.007 mg/mL). Then, when different surfactants and co-surfactants were tested, IR had a satisfying solubility in T-80 (0.671 ± 0.018 mg/mL) and PEG-400 (0.696 ± 0.014 mg/mL). However, PEG-400 and T-80 were not mutually miscible, resulting in delamination in a few minutes. Therefore, Cremophor EL (0.588 ± 0.006 mg/mL) was selected as the surfactant for further study. In brief, MCT was selected as the oil phase, Cremophor EL as the surfactant, and PEG-400 as the co-surfactant to develop the IR-ME system.

#### 2.1.2. Determination of Efficient ME Region

A pseudo-ternary phase diagram was constructed to calculate the region of the micro-emulsion region when the mass ratio of surfactant versus co-surfactant was calculated as 4:1, 3:1, 2:1, 1:1, 1:2, and 1:3. Then, the mixed surfactant (surfactant mixture; Smix) was prepared and the self-microemulsifying region was determined. The microemulsion area was large, which indicated that the ME formulation was stable. In addition, with increasing surfactant concentration, the self-microemulsifying region was increased, and the largest emulsification region was found at the ratio of 4:1 (Figure 2B). The increased interfacial fluidity, entropy, and decreased interfacial tension might be a possible explanation [24]. Then, according to the mass ratio of oil phase and Smix, the microemulsion was prepared at the ratio of 9:1~1:9 (Km). The results showed that the Km of the oil phase and mixed emulsifier was 1:9 and 2:8 displayed with a clear and transparent state. The remaining ratios changed from clarification to turbidity. Comprehensive evaluation of the color and the amount of surfactants was performed, and the ratio of the oil phase to the mixed surfactant was determined as 2:8.

#### 2.1.3. Determination of Water Content of ME

The conductivity value of the ME was measured using a conductivity meter [25,26]. The conductivity of ME with different water contents were measured and three trends were observed (Figure 2C): when the water content increased from 10% to 60%, the conductivity of ME showed a rapid increase trend; when the water content was 60–68%, the increase trend was flat. When the water content was 68–90%, the conductivity decreased rapidly. According to the above results, it can be divided into three regions, representing the w/o type, the bicontinuous phase, and the o/w type, respectively. With the conductivity of the solution gradually decreasing, the peak was the critical point of w/o. According to the conductivity method, the optimum water content of the microemulsion was 68%.

#### 2.1.4. Preparation of IR-ME Gel

However, the fluidity, aggregation, and drug precipitation during storage of ME highly limited the application to the skin. Therefore, ME gel was prepared to reduce the fluidity of the emulsion, increase the adhesion ability, and improve the absorption and bioavailability of the drug. Carbomer 934 is a polymer compound formed by cross-linking of acrylic acid and acryloyl sucrose, which belongs to the water-based gel in the single-phase gel. It is spreadable, non-greasy, easy to clean, comfortable to use, and lubricating, making it suitable for topical delivery systems [27].

### 2.2. Characterization of IR-ME and IR-ME Gel

#### 2.2.1. Fourier-Transform Infrared (FT-IR) Analysis

**Figure 3:**
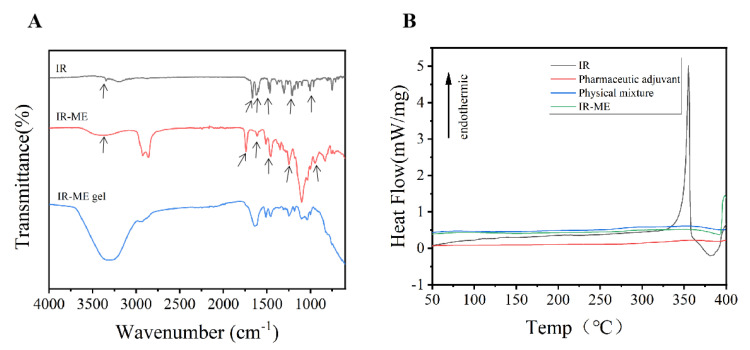
Drug and adjuvants compatibility. (**A**) FTIR spectra of IR, IR-ME, and IR-ME gel (arrows indicate the characteristic infrared absorption peak of IR). (**B**) DSC thermograms of IR, Pharmacevtic adjuvant, physical mixture and IR-ME.

FT-IR analysis was performed to investigate the chemical structures of drug molecules in ME and ME gel (Figure 3A). In the FTIR spectrum, peaks were observed at 3345 cm^−1^ due to N-H stretch: 3346 cm^−1^ due to the stretching vibration of the chelation structure of C=O and hydrogen bonds, and 3230–2857 cm^−1^ due to C–H stretch. Stretching vibration of the conjugated system of C=C, C=O, and N–H groups was observed at 1703–1595 cm^−1^ and 1481 and 1464 cm^−1^ corresponding to the ring C–C stretching bands. The region below 1381 cm^−1^ was due to the deformation vibration of N–H and C–H [28]. The results showed that there was no obvious deviation of the above absorption peaks in ME, and the main peak of IR will not be greatly shifted due to adjuvants, indicating that ME does not produce chemical changes to drug molecules. The drug and ME are primarily physically bound. However, the spectrum of ME gel hardly represents the corresponding drug peaks, which may be due to the dense hydrogel framework of carbomer 934.

#### 2.2.2. Differential Scanning Calorimetry (DSC) Analysis

The analytical DSC was adopted to determine the thermal properties (melting points, phase transitions) of drugs and adjuvants. Differential scanning calorimetry maps among different groups are shown in Figure 3B. The endothermic value of 355.3 °C was the crystal endothermic peak of IR, and the adjuvants showed a constant temperature. There were no such peaks in both ME and physical mixture spectra. The reason for the analysis may be that the IR crystals were melted in the molten carrier without showing the crystal endothermic peak.

#### 2.2.3. TEM and SEM Analysis

**Figure 4:**
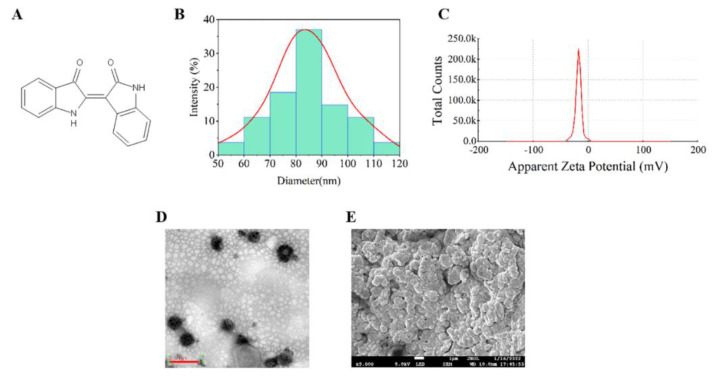
Physicochemical characterization of IR-loaded ME and IR-loaded ME-based hydrogel. (**A**) IR chemical structure. (**B**) Size distribution of the IR-loaded ME. (**C**) The zeta potential distribution of IR-loaded ME. (**D**,**E**) The electron micrographs of ME and ME in ME gel (Scale bar: 0.2 μm).

The particle size is an important factor for the successful preparation of ME formulations, as it determines the smooth release and penetration of the formulation through the epidermis. Nanoscale droplets help penetrate the multi-layered skin structure and provide a larger surface area for faster drug release [29]. Furthermore, Kotta et al. [30] reported that the presence of a large amount of Smix in ME is beneficial for reducing the liquid size. The polydispersity index (PDI) is also a measure that demonstrates the uniformity of droplets in a formulation. A PDI closer to zero indicates more uniform nanoparticles. Furthermore, the negative charge on the surface of ME droplets is beneficial to help the droplets penetrate the skin. Therefore, this study characterized the above factors (Figure 4A–D). The average particle size of the prepared IR-loaded ME was 84.37 nm, and PDI < 0.2, and zeta potential were in the range of 0~−20 mV. The SEM of ME gel is shown in Figure 4E.

### 2.3. In Vitro Skin Permeation and Deposition Study

**Figure 5:**
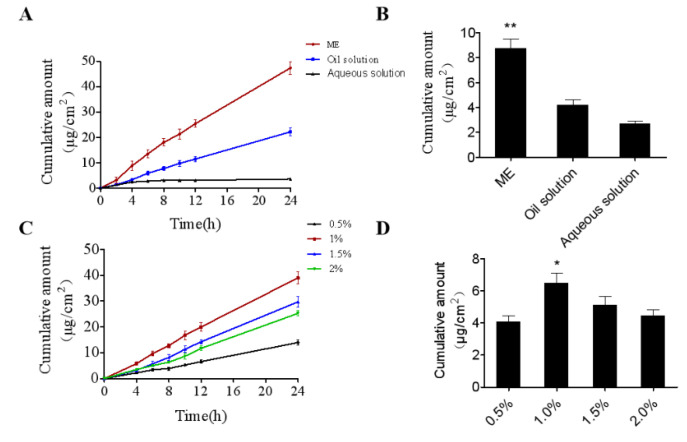
In vitro transdermal permeation profiles. (**A**) The vitro transdermal release curve of IR in different carriers. (**B**) The retention amount of IR in the skin of different carriers. (**C**) The vitro transdermal release curve of IR in different substrate concentrations. (**D**) The retention amount of IR in the skin of different substrate concentrations. The results were expressed as the mean ± SEM. The comparisons between groups were performed using one-way ANOVA (Tukey’s multiple comparisons test). ** *p* < 0.01 vs. Oil solution and Aq solution; * *p* < 0.05 vs. 0.5%, 1.5% and 2% gel (*n* = 3).

Franz diffusion cells were established to assay the in vitro penetration behavior of ME, ME gel, medicinal olive oil solution, and aqueous solution. Steady-state transdermal flux was determined from the curve of cumulative drug penetration versus time. After 24 h, the cumulative permeability of ME, oil solution, and aqueous solution were 47.34 ± 3.59 μg/cm^2^, 22.21 ± 2.14 μg/cm^2^, 3.60 ± 0.11 μg/cm^2^, and the skin retention was 8.77 ± 1.26 μg/cm^2^, 4.18 ± 0.79 μg/cm^2^, and 2.68 ± 0.36 μg/cm^2^, respectively (Figure 5A,B). The results showed that the accumulated drug exudation in ME was 2.1-fold and 13.1-fold higher than that of the oil solution and the aqueous solution, respectively; both the permeation and retention of the skin increased. The reason for the analysis may be that the particle size of ME is small and interacts with the stratum corneum, which ultimately improves the permeability of the drug. In addition, the optimal gel concentration of ME was 1%, and the transdermal flux and skin retention at 24 h were 39.01 ± 3.21 μg/cm^2^ and 6.5 ± 0.6 μg/cm^2^, respectively (Figure 5C,D). Table 1 lists the in vitro penetration parameters among different formulations of IR through mouse dorsal skin. The results showed that the transdermal flux of ME gel was lower than that of ME, which may be due to the high viscosity of the gel itself. Despite the reduced flux of ME gel, it is still favored for their long-lasting effect, ease of application to the skin, and ease of cleaning.

### 2.4. In Vivo Studies

#### 2.4.1. Macroscopic Observation of Dorsal Tissue

**Figure 6:**
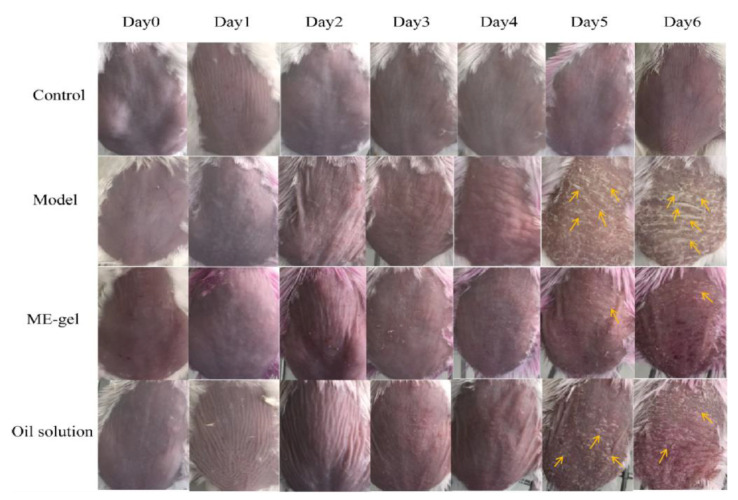
The back psoriasis-like symptoms of experimental mice in each group were evaluated after 6 days of topical application of ME gel and oil solution (arrows indicate the silvery scales of psoriasis skin and after treatment).

**Figure 7:**
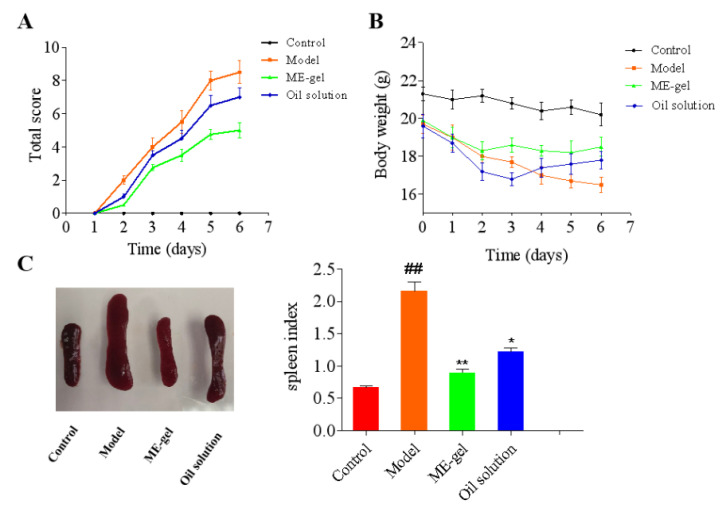
The therapeutic effect of IR. (**A**) PASI score of indirubin on the back skin of IMQ-induced mice. (**B**) Effects of IR on body weight change of IMQ-induced mice. (**C**) Effects of IR on spleen index of IMQ-induced mice. The results were expressed as the mean ± SEM. The comparisons between groups were performed using one-way ANOVA (Tukey’s multiple comparisons test). * *p* < 0.05 and ** *p* < 0.01 vs. Model; ## *p* < 0.01 vs. Control (*n* = 6).

Imiquimod (IMQ) cream was applied to develop psoriasis-like skin features on the back of mice (Figure 6). From the third day onwards, the degree of skin lesions in the model group increased sharply, scale accumulation appeared, and the skin felt thick and hard. Compared with the model group and the oil solution group, ME gel treatment significantly reduced the scales, erythema, and thickening of the skin; the surface was smooth and flat, and there was basically no scaling. After treatment of the formula on the established IMQ model, each group of mice was scored using corrected psoriasis area and severity index (PASI) score, as shown in Figure 7A. These results suggested that ME gel has good therapeutic activity, and the efficacy was better than the oil solution group.

#### 2.4.2. Body Weight and Spleen Index of Mice 

The spleen is the largest immune organ in humans and animals, and it is also the center of cellular and humoral immunity, containing a large number of immune cells. When the body suffers from an inflammatory disease, the spleen undergoes changes to a certain extent, and the spleen index is an important sign of an immune disease. Therefore, the therapeutic effect of drugs on inflammatory diseases can be judged by changes in the color and the spleen index. The body weight of the mice in the model group continued to decrease until it eased slightly on the 6th day (the symptoms began to subside on the 6th day after modeling). After drug treatment, the weight loss trend of mice was reduced on the third day (Figure 7B). After the mice were modeled, the immune system was stimulated by a large amount of IMQ ointment, resulting in an enlarged and blackened spleen and increased spleen index. As shown in Figure 7C, the spleen index of mice in the model group was significantly larger than that of control mice. After the drug treatment, the spleen index of the mice decreased, and especially the ME gel treatment group had a significant difference compared with the model group.

#### 2.4.3. Skin Histopathology

**Figure 8:**
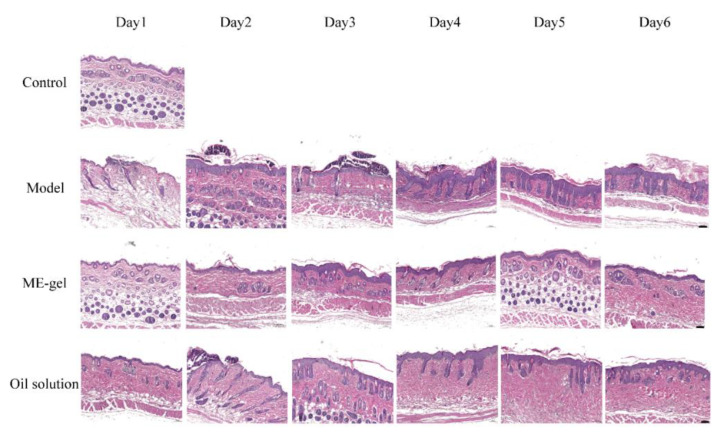
Effect of IR on skin cytokines in psoriatic mice (magnification 100×; scale bar 100 µm).

The anti-psoriatic effect of ME gel formulation was observed by H&E staining. The histological changes of different groups of skin specimens are shown in Figure 8. The epidermal layer of mice in the model group was significantly thickened, and there was obvious parakeratosis and hyperkeratosis, regular downward extension of the epidermis, high-density anucleated cells and a continuous granular layer, a large amount of inflammatory cell infiltration, and Munro microabscesses. These are typical of human psoriatic skin. The olive oil group showed that the thickening of the epidermis and acanthosis was reduced, but the regular downward protrusion of the epidermis still existed. The effect of ME gel in reducing epidermal hyperplasia, hyperkeratosis, and acanthosis on day six was very similar to that of the control group.

#### 2.4.4. Immunohistochemical

**Figure 9:**
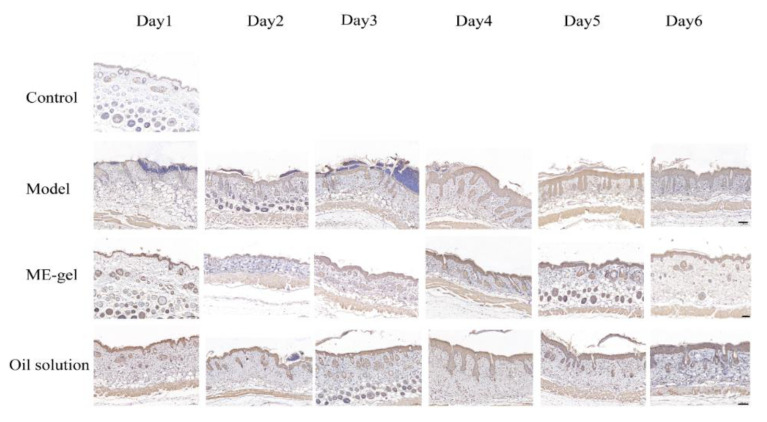
Effects of IR on the expression of IL-17 on the back skin of psoriatic mice (magnification 100×; scale bar 100 µm).

**Figure 10:**
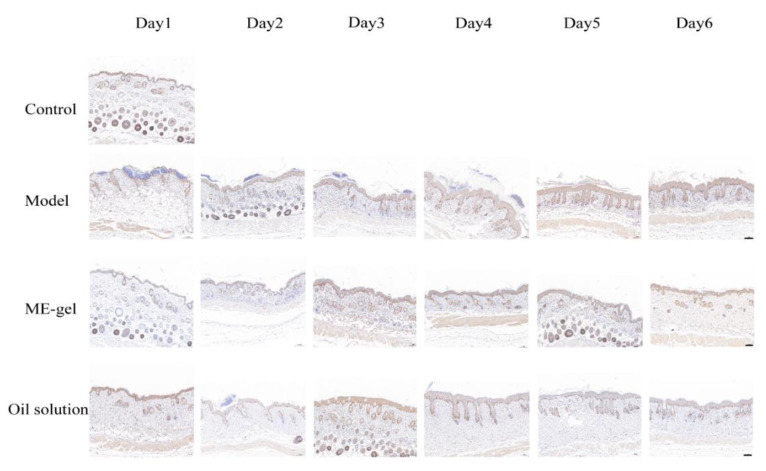
Effects of IR on the expression of Ki67 on the back skin of psoriatic mice (magnification 100×; scale bar 100 µm).

**Figure 11:**
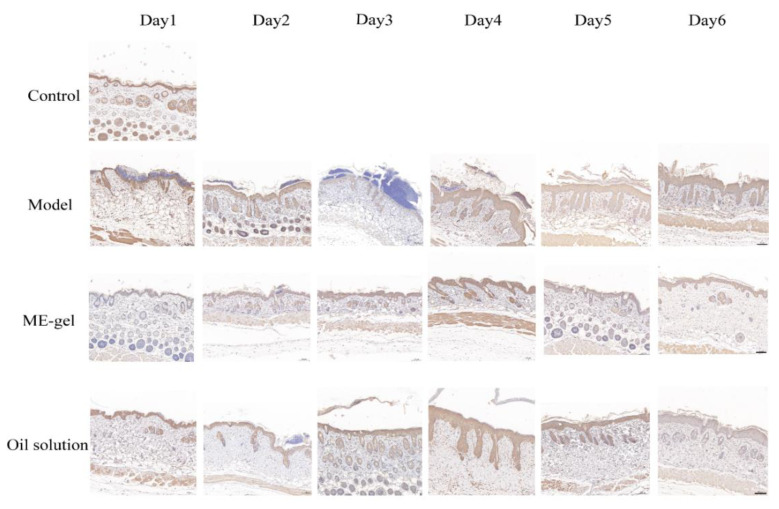
Effects of IR on the expression of CD4^+^T on the back skin of psoriatic mice (magnification 100×; scale bar 100 µm).

Dysregulation of the IL-23/IL-17 cytokine axis has been reported to be central to the pathogenesis of human psoriasis. Il-23 and IL-17 play an important role in the pathogenesis of psoriasis by affecting T-Helper 17 (Th17) [31]. Th17 cells produce cytokines such as IL-21, IL-17, and IL-22 to accelerate psoriasis symptoms. In addition, Ki67 is a nuclear protein closely related to cell proliferation, and is usually used as a marker protein for detecting cell proliferation. By detecting the expression of Ki67 in disease-like skin, the proliferation state of the skin can be judged. Furthermore, CD4^+^T cells are important immune cells in the pathogenesis of psoriasis. Under the stimulation of various inflammatory factors, they can differentiate into Th17 cells, Th22 cells, and other helper T cells to promote the inflammatory response of psoriasis. Therefore, the present study analyzed the levels of key cytokines IL-17A (Figure 9), Ki67 (Figure 10), and CD4^+^T (Figure 11). With the change of time, the expression levels of IL-17A, Ki67, and CD4^+^T in the model group were significantly increased. Compared with the model group, the expression levels of IL-17A, Ki67, and CD4^+^T in the ME gel group were significantly decreased on the sixth day. The decrease of IL-17 suggests that the therapeutic effect of IR on psoriasis mouse model may be related to the intervention of IL-23/IL-17 inflammatory axis. Ki67 staining demonstrated that hyperproliferation of epidermal cells was inhibited, possibly due to the immunomodulatory effects of IR. In addition, the decreased portion of CD4^+^T-positive cells indicates that ME gel could alleviate psoriasis disorders via immune regulation.

## 3. Discussion

The self-microemulsion drug delivery system (SMEDDS) is a new type of transdermal drug delivery system with great potential [32]. Due to the low interfacial tension of ME, it can be formed spontaneously under certain conditions. Due to the fluidity of the interfacial film, the drug can be freely transported between the phases. The transdermal mechanisms of ME include: improving the solubility and loading of drugs, increasing the hydration of the stratum corneum, and destroying the lipid bilayer of the stratum corneum. The basic composition of self-microemulsion includes an oil phase, surfactant, and co-surfactant. When selecting preparation adjuvants, it is necessary to fully consider the compatibility between adjuvants, the compatibility of adjuvants and drugs, and the toxicity and irritation of adjuvants to ensure that the selected self-microemulsion components are non-toxic, non-irritating, and do not affect drug efficacy [26]. Adjuvants having a high dissolving ability to the drug is particularly important for the preparation of self-microemulsion. Therefore, in view of the properties and characteristics of IR, a suitable oil phase, emulsifier, co-emulsifier, and the ratio of each auxiliary material are key for the successful preparation of self-microemulsions. In this study, we found that appropriate surfactants and co-surfactants could significantly improve the solubility and stability of drugs. ME can enhance drug penetration by dilating sweat glands and hair follicles through the permeation-enhancing effect of surfactants and co-surfactants. In addition, surface, particle size and charge are important characteristics of ME in transdermal drug delivery. For example, a PDI less than 0.2 is a key parameter to ensure an even distribution of the ME [33]. In addition, previous reports have shown that when the particle size of ME is greater than 600 nm, the drug mainly stays in the stratum corneum and cannot be delivered to the deep layers of the skin. However, while the particle size is less than 300 nm, it can be effectively delivered to the deep layers of the skin. In addition, the high surface charge prevents particle aggregation, resulting in good stability [34].

However, as a liquid preparation, ME has the characteristics of strong fluidity, short residence time, and poor adhesion. These shortcomings limit the practical applications of ME. Therefore, in order to create a formulation with a certain adhesion ability, a hydrogel thickener was adopted in this experiment. As a transdermal drug delivery carrier, ME gel concentrates the characteristics of the gel’s adhesion to the skin, the deformation fluidity under the action of external force, and the complete dispersibility to the drug, which keeps the drug spreading evenly and in the lesion for a long time. This finding also suggest that ME could walk freely in the hydrogel network and successfully deliver drugs to the stratum corneum before penetrating and releasing the drug into the deeper layers of the skin. Appropriate viscosity could ensure good adhesion of the hydrogel to the skin. However, high gel viscosity could impede the diffusion of the drug to the skin, probably due to its dense three-dimensional network structure. Therefore, it is important to choose an appropriate matrix concentration. Topical application of ME gel has proven to be an essential option for the treatment of skin diseases such as psoriasis, avoiding the first-pass metabolism associated with oral doses and increasing the drug concentration of drug targets. In addition, the versatility and ease of preparation offer tremendous scalability for industrial companies. However, several limitations still exist, such as a lack of comparison with known drugs for the treatment of psoriasis in this study.

In conclusion, ME gel can be used as a transdermal drug delivery carrier to increase the transdermal flow of drugs, release high doses of drugs for a long time, and reduce skin irritation, thereby achieving the purpose of improving drug efficacy and safety. Therefore, ME gel has unique advantages in the field of transdermal drug delivery systems.

## 4. Materials and Methods

### 4.1. Materials

IR (purity ≥ 98%) was synthesized in the laboratory of Huaqiao University. Olive oil, castor oil polyoxyethylene ether, and Tween 80 glycerin were purchased from Shanghai Rin en Technology Development Co., Ltd. (Shanghai, China). Sino chain triglyceride was purchased from Beijing Ita Biotechnology Co., Ltd. (Beijing, China). Diethylene glycol monoethyl ether was purchased from Shanghai Bide Medical Technology Co., Ltd. (Shanghai, China). Kapom was purchased from Shanghai Maclean Biochemical Technology Co., Ltd. (Shanghai, China). Ethanol and other chemical reagents were purchased from China Pharmaceutical Group Co., Ltd. (Beijing, China). 5% IMQ cream was purchased from Sichuan Mingxin Pharmaceutical Co., Ltd. (Chengdu, China). Other chemicals were analytical grade or chromatographic grade. 

### 4.2. IR Detection Method

The concentration of IR was determined by the high performance liquid chromatography (HPLC) method, and the chromatographic condition was shown as below. Column: Diamonsil C18 column (250 mm × 4.6 mm, 5 μm); Mobile phase: methanol/water (70:30); Flow rate: 1.0 mL/min; Detection wavelength: 292 nm; Column temperature: 40 °C; Injection volume: 20 μL. Taking the mass concentration of IR as the abscissa (C) and the peak area as the ordinate (A) to draw the standard curve, the linear regression equations are: A = 34,156C − 6356.4 (R^2^ = 0.9993). The results showed that indirubin had a good linear relationship in the concentration range of 0.5–20 µg/mL.

### 4.3. Preparation of IR-ME and IR-ME Gel

The solubility of IR in different phases was analyzed. At room temperature, 2 mL of solvent (oil phase, surfactant, cosurfactant) was added into the EP tube with a capacity of 5 mL, and the excess IR was added and vortexed for dispersion. After shaking at room temperature for 48 h to reach solution equilibrium, centrifuging was performed at 6000 g/min for 15 min. Then, the supernatant was collected, diluted, and analyzed by a UV-vis spectrophotometer at UV-absorption wavelength of 535 nm. Each sample was tested three times.

Medium chain triglyceride was selected as the oil phase; castor oil polyoxyethylene ether was selected as the surfactant; PEG-400 was selected as the emulsifier. First, according to the mass ratio of surfactant versus co-surfactant of 4:1, 3:1, 2:1, 1:1, 1:2, and 1:3, the mixed surfactants (surfactant mixture; Smix) were prepared. Then, the oil phase and Smix were mixed at room temperature at varying weight ratios of 1:9 to 9:1 %*w*/*v* to characterize phase boundaries. The mixtures were slowly titrated with distilled water as the aqueous phase and stirred until equilibrium (800 rpm). Under the ratio, the solution changed from clear to cloudy or from cloudy to clear, and the volume of water at the end point of the titration was recorded. Pseudo-ternary phase diagrams were drawn with oil phase, Smix, and distilled water as three vertices, respectively. Origin 2021 software was used to create a pseudo-ternary phase diagram and determine the optimal scale according to the size of the pseudo-ternary phase diagram. The IR was then added to the ME system.

A certain amount of Carbomer 934 was weighed and dispersed in water such that its final concentration was 0.5%, 1%, 1.5%, and 2%, respectively. Then, 10% glycerol was added as a humectant, and left overnight to fully swell it. Afterwards, 20% triethanolamine was added dropwise after swelling to adjust the pH value to 5.5~6.5. In the end, an appropriate amount of microemulsion was added to the blank matrix and stirred at 1000 rpm for 10 min to obtain the IR-ME gel.

### 4.4. Characterization of IR-ME and IR-ME Gel

Infrared spectra (FT-IR) of IR, ME and ME gel were recorded with a Nicolet IS50 FT-IR spectrometer (Thermo Fisher Scientific, Waltham, MA, USA) with a range of 4000–400 cm^−1^ and a resolution of 4 cm^−1^.

Heat maps of indirubin, adjuvants, physical mixtures, and ME were recorded using a Netzsch DSC214 differential scanning calorimeter (Netzsch, Germany). Each sample was sealed in an aluminum crucible and heated to 400 °C from room temperature at a rate of 10 °C·min^−1^ under nitrogen.

The morphology of the microemulsion was observed by FEI TECNAI G2 F20 (TEM) (FEI, Hillsboro, OR, USA) at an accelerating voltage of 100 kV. A few samples were dropped onto the copper mesh, wetted for several minutes, stained with 2% phosphotungstic acid staining solution for 5 min, dried at room temperature, and observed by TEM.

The morphology and surface characteristics of ME gel were detected by JEOL JSM-7800F scanning electron microscope (SEM) (JEOL Ltd., Tokyo, Japan). The ME gel was coated on the tin foil, vacuumed and adhered to the copper plate with double-sided tape, and sprayed with gold under reduced pressure, and the morphological structure of the sample was observed under the scanning electron microscope.

### 4.5. In Vitro Transdermal Test

SPF BALB/c female mice (6–8 weeks; body weight 18–22 g) were purchased from Shanghai Slack Laboratory Animal Co., Ltd. (Shanghai, China). All animal testing protocols were approved by the Animal Care and Use Committee of Huaqiao University, and all experimental procedures were in accordance with the National Institutes of Health Guide for the Care and Use of Laboratory Animals. All mice were placed in an environmental control room with a temperature of 22 ± 2.0 °C, a light-dark cycle of 12 h, and ate freely.

#### 4.5.1. Preparation of Excised Skin

Mice were sacrificed by cervical vertebrae, and the back hair was shaved with an electric shaver. Then an appropriate amount of depilatory cream was applied, and washed off with water after one to two minutes. The skin on the back of the mouse was excised, removed subcutaneous tissue and fat, then rinsed with normal saline. The retrieved samples were subsequently stored at −20 °C and used within 1 week. Prior to the experiment, isolated mouse skin was removed, naturally thawed to room temperature, tested for skin integrity and rinsed with saline.

#### 4.5.2. In Vitro Transdermal Test

In the present study, the transdermal diffusion of ME, ME gel, and olive oil solution in vitro was investigated by Franz diffusion cells. The mouse skin was fixed between the two halves of the diffusion cell, with the epidermis facing the donor compartment and the dermis facing the receptor compartment and in close contact with the receiving fluid. The volume of the receiving tank was 10 mL, and the effective area of the diffusion tank was 0.8 cm^2^. The receiving solution was a saline solution containing 30% ethanol, and the receiving solution was degassed by ultrasonic waves at the same time. The temperature of the constant temperature water bath was 35 °C, and the rotation speed was 500 rpm. After 24 h, the skin was removed, and the effective skin through which the drug penetrated was carefully cut off and rinsed with normal saline. The skin samples were minced, 1 mL of DMF was added for sonication for 1 h, the above steps were repeated once. The two supernatants were combined, filtered through a 0.22 μm microporous membrane, and injected into a high-performance liquid chromatography analyzer. The amount of IR retained in the formulation was calculated after 24 h. At the same time, samples were taken from the receiving medium to study the transdermal accumulation of the drug.

### 4.6. In Vivo Studies

#### 4.6.1. Experimental Design

Twenty-four female BALB/C mice were randomly divided into 4 groups: control group, model group, ME gel group, and olive oil solution group, with 6 mice in each group. One week after acclimatization, both sides of the back and spine of mice were depilated 24 h before the experiment. Psoriasis model was induced on the mice skin by the application of IMQ cream as a dose of 62.5 mg/day/mouse for a period of 6 days. Mice in the intervention group were given corresponding drugs at 4 and 8 h after IMQ induction.

#### 4.6.2. Assessment of Psoriasis-like Symptoms

The skin thickness, erythema, and scale of the back skin of the experimental mice were scored using the corrected PASI score, and the severity of inflammation was observed. According to skin thickness, severity of erythema and scaling, a scale of 0 to 4 was used, namely 0, asymptomatic; 1, slight; 2, moderate; 3, severe; 4, very severe. Scoring was performed on each day of the experimental period.

#### 4.6.3. Histopathology

The purpose of this study was to investigate the pathological changes in mouse skin after applying each preparation. Mice were sacrificed six days later, and skin samples were collected and immersed in 4% paraformaldehyde for fixation. Then tissue paraffin sections were made, the tissues were stained with hematoxylin and eosin, and the histopathological changes of each experimental group were observed under an inverted microscope.

#### 4.6.4. Immunohistochemistry

First, the paraffin sections were dried in a 60 °C oven for 30 min to melt the wax, and then placed in xylene-gradient ethanol-water for dewaxing and immersion; hydrogen peroxide was added to eliminate endogenous peroxidase; then, antigen retrieval solution was used to repair antigens. After permeabilization and blocking, the diluted primary antibody was added for incubation, the primary antibody was washed off, and the secondary antibody was added to incubate at room temperature. After that, DAB was used for color development, hematoxylin staining was used, and the results were observed and recorded.

### 4.7. Statistical Analysis

Experiments were performed in triplicate and data were expressed as mean ± standard deviation. Statistical analysis of the acquired data was performed using GraphPad Prism software version 5.0. Analysis of variance (ANOVA) with Tukey method as a post-hoc test and Student’s *t*-tests were used to analyze the data. These differences were considered statistically significant at *p* < 0.05.

## 5. Conclusions

The poor solubility and bioavailability limited the application of IR. Herein, to improve the skin permeability, the ME system was adopted to the local delivery of IR. The ME was physically stable, nano-sized, spherical, and with a narrow-size distribution. Moreover, the ME gel highly improved the permeation rate in vitro and alleviated psoriasis disorders induced by IMQ. This study suggests that IR-loaded ME formulation could be used for topical administration in the treatment of psoriasis or other topical skin disorders. In summary, this study presents a new direction for the treatment of psoriasis with limited solubility and transdermal efficiency.

## Figures and Tables

**Table 1:** In vitro permeation parameters of IR from different formulations through mice back skin (*n* = 3).

Parameter	Q ^a^	Jss ^b^	Q (Skin) ^c^
ME	47.34 ± 3.59	1.96 ± 0.08	8.77 ± 1.26
ME gel (1%)	39.01 ± 3.21	1.65 ± 0.12	6.5 ± 0.6
Oil solution	22.21 ± 2.14	0.94 ± 0.09	4.18 ± 0.79
Aqueous solution	3.60 ± 0.11	0.05 ± 0.01	2.68 ± 0.36

^a^ Cumulative amount of IR permeated (µg·cm^−2^) after 24 h. ^b^ Flux (permeation rate constant) at steady state (µg·cm^−2^·h^−1^), obtained from the slope of the regression line after plotting the amount of IR permeated vs. time. ^c^ Amount deposited in the skin after 24 h (µg·cm^−2^).

## Data Availability

The data generated during the current study are available with the corresponding author on reasonable request.

## References

[1] Bieber T. (2021). AhR Modulating Agent for the Topical Therapy of Plaque Psoriasis. N. Engl. J. Med..

[2] Georgescu S.-R., Tampa M., Caruntu C., Sarbu M.-I., Mitran C.-I., Mitran M.-I., Matei C., Constantin C., Neagu M. (2019). Advances in Understanding the Immunological Pathways in Psoriasis. Int. J. Mol. Sci..

[3] Griffiths C.E.M., Armstrong A.W., Gudjonsson J.E., Barker J.N.W.N. (2021). Psoriasis. Lancet.

[4] Prinz I., Sandrock I., Mrowietz U. (2020). Interleukin-17 cytokines: Effectors and targets in psoriasis-A breakthrough in understanding and treatment. J. Exp. Med..

[5] Benezeder T., Wolf P. (2019). Resolution of plaque-type psoriasis: What is left behind (and reinitiates the disease). Semin. Immunopathol..

[6] Said J., Elman S., Perez-Chada L., Mita C., Merola J., LeBoeuf N. (2022). Treatment of Immune Checkpoint Inhibitor-Mediated Psoriasis: A Systematic Review. J. Am. Acad. Dermatol..

[7] Rapalli V., Waghule T., Gorantla S., Dubey S., Saha R., Singhvi G. (2020). Psoriasis: Pathological mechanisms, current pharmacological therapies, and emerging drug delivery systems. Drug Discov. Today.

[8] Huang T., Lin C., Alalaiwe A., Yang S., Fang J. (2019). Apoptotic or Antiproliferative Activity of Natural Products against Keratinocytes for the Treatment of Psoriasis. Int. J. Mol. Sci..

[9] Bonesi M., Loizzo M., Provenzano E., Menichini F., Tundis R. (2016). Anti-Psoriasis Agents from Natural Plant Sources. Curr. Med. Chem..

[10] Gamret A., Price A., Fertig R., Lev-Tov H., Nichols A. (2018). Complementary and Alternative Medicine Therapies for Psoriasis: A Systematic Review. JAMA Dermatol..

[11] Gaitanis G., Magiatis P., Velegraki A., Bassukas I.D. (2018). A traditional Chinese remedy points to a natural skin habitat: Indirubin (indigo naturalis) for psoriasis and the Malassezia metabolome. Br. J. Dermatol..

[12] Xie X.-J., Di T.-T., Wang Y., Wang M.-X., Meng Y.-J., Lin Y., Xu X.-L., Li P., Zhao J.-X. (2018). Indirubin ameliorates imiquimod-induced psoriasis-like skin lesions in mice by inhibiting inflammatory responses mediated by IL-17A-producing γδ T cells. Mol. Immunol..

[13] Lin Y.K., See L.C., Huang Y.H., Chi C.C., Hui R.C. (2018). Comparison of indirubin concentrations in indigo naturalis ointment for psoriasis treatment: A randomized, double-blind, dosage-controlled trial. Br. J. Dermatol..

[14] Gaboriaud-Kolar N., Vougogiannopoulou K., Skaltsounis A. (2015). Indirubin derivatives: A patent review (2010–present). Expert Opin. Ther. Pat..

[15] Rahman M., Akhter S., Beg S. (2018). Nanomedicine Advances in Topical Infective and Non-Infective Skin Diseases Therapy. Recent Pat. Anti-Infect. Drug Discov..

[16] Saleem S., Iqubal M., Garg S., Ali J., Baboota S. (2020). Trends in nanotechnology-based delivery systems for dermal targeting of drugs: An enticing approach to offset psoriasis. Expert Opin. Drug Deliv..

[17] Hoffman M., Hill D., Feldman S. (2016). Current challenges and emerging drug delivery strategies for the treatment of psoriasis. Expert Opin. Drug Deliv..

[18] Hu Q., Lin H., Wang Y., Wang X., Yao J., Fu X., Yu X. (2021). Design, optimization and evaluation of a microemulsion-based hydrogel with high malleability for enhanced transdermal delivery of levamisole. Int. J. Pharm..

[19] Li F., Shen J., Bi J., Tian H., Jin Y., Wang Y., Yang X., Yang Z., Kou J. (2016). Preparation and evaluation of a self-nanoemulsifying drug delivery system loaded with Akebia saponin D-phospholipid complex. Int. J. Nanomed..

[20] Salimi A., Sharif Makhmal Zadeh B., Hemati A., Akbari Birgani S. (2014). Design and Evaluation of Self-Emulsifying Drug Delivery System (SEDDS) Of Carvedilol to Improve the Oral Absorption. Jundishapur J. Nat. Pharm. Prod..

[21] Jain S., Patel N., Shah M., Khatri P., Vora N. (2017). Recent Advances in Lipid-Based Vesicles and Particulate Carriers for Topical and Transdermal Application. J. Pharm. Sci..

[22] Kováčik A., Kopečná M., Vávrová K. (2020). Permeation enhancers in transdermal drug delivery: Benefits and limitations. Expert Opin. Drug Deliv..

[23] Jagdale S., Deore G., Chabukswar A. (2018). Development of Microemulsion Based Nabumetone Transdermal Delivery for Treatment of Arthritis. Recent Pat. Drug Deliv. Formul..

[24] Alvarado H.L., Abrego G., Souto E.B., Garduño-Ramirez M.L., Clares B., García M.L., Calpena A.C. (2015). Nanoemulsions for dermal controlled release of oleanolic and ursolic acids: In vitro, ex vivo and in vivo characterization. Colloids Surf. B Biointerfaces.

[25] Pajić N.Z.B., Todosijević M.N., Vuleta G.M., Cekic N., Dobričić V.D., Vučen S.R., Čalija B.R., Lukic M., Ilić T.M., Savic S. (2017). Alkyl polyglucoside vs. ethoxylated surfactant-based microemulsions as vehicles for two poorly water-soluble drugs: Physicochemical characterization and in vivo skin performance. Acta Pharm..

[26] Szumała P., Macierzanka A. (2022). Topical delivery of pharmaceutical and cosmetic macromolecules using microemulsion systems. Int. J. Pharm..

[27] Choudhury H., Gorain B., Pandey M., Chatterjee L.A., Sengupta P., Das A., Molugulu N., Kesharwani P. (2017). Recent Update on Nanoemulgel as Topical Drug Delivery System. J. Pharm. Sci..

[28] Ju Z., Sun J., Liu Y. (2019). Molecular Structures and Spectral Properties of Natural Indigo and Indirubin: Experimental and DFT Studies. Molecules.

[29] Jangdey M., Gupta A., Saraf S. (2017). Fabrication, in-vitro characterization, and enhanced in-vivo evaluation of carbopol-based nanoemulsion gel of apigenin for UV-induced skin carcinoma. Drug Deliv..

[30] Kotta S., Khan A., Ansari S., Sharma R., Ali J. (2015). Formulation of nanoemulsion: A comparison between phase inversion composition method and high-pressure homogenization method. Drug Deliv..

[31] Van der Fits L., Mourits S., Voerman J.S.A., Kant M., Boon L., Laman J.D., Cornelissen F., Mus A.-M., Florencia E., Prens E.P. (2009). Imiquimod-induced psoriasis-like skin inflammation in mice is mediated via the IL-23/IL-17 axis. J. Immunol..

[32] Li F., Hu R., Wang B., Gui Y., Cheng G., Gao S., Ye L., Tang J. (2017). Self-microemulsifying drug delivery system for improving the bioavailability of huperzine A by lymphatic uptake. Acta Pharm. Sin. B.

[33] Guo Y., Mao X., Zhang J., Sun P., Wang H., Zhang Y., Ma Y., Xu S., Lv R., Liu X. (2019). Oral delivery of lycopene-loaded microemulsion for brain-targeting: Preparation, characterization, pharmacokinetic evaluation and tissue distribution. Drug Deliv..

[34] Gharib R., Auezova L., Charcosset C., Greige-Gerges H. (2017). Drug-in-cyclodextrin-in-liposomes as a carrier system for volatile essential oil components: Application to anethole. Food Chem..

